# 

**Published:** 1882-11

**Authors:** 


### Ohio State Dental Society.

The seventeenth annual meeting of this society will be held in the city of Columbus, on the first Wednesday of December next, at 10 o'clock A.M.

The following programme of subjects has been prepared, and will be presented for consideration:

1st. Mechanical Dentistry—the relative merits of the different materials for bases of artificial teeth and their effects upon the tissues.

2d. Best Methods of Filling Roots.

3d. Plastic Fillings and their Availability.

4th. Dental Periostitis.

5th. Oral Surgery.

Upon each of these subjects it is expected that there will be one or more papers, and it is very desirable that every member should come prepared to give his opinions and experiences upon each of these subjects, and so far as practicable bring models and illustrations.

The society is in a very prosperous condition. Financially it stands well. It has an active membership of one hundred and six, honorary and associate members twenty, and, notwithstanding this goodly number, there is still room.

There are fully two hundred members of the profession in the State in addition to the present membership, who ought to be in the society. The society needs them and they need the society, and the best interest of their patients requires that they should avail themselves of this and similar opportunities for improvement.

Now, let every dentist, who thinks these statements true, be present, and this society will have such a meeting as it has not hitherto experienced.

There will be a number of matters important to the profession in addition to the programme already given that will come up for consideration.

These miscellaneous subjects are often of more striking interest than the announced subjects.

The day when men can sustain themselves in efficient practice in a state of isolation and separation from their brethren has passed by, and in these days no man can occupy an isolated position without the protest of his own sense of propriety and right. Then let the dental profession of the State of Ohio come together on the first Wednesday of December next, in its strength and earnestness.

### Examinations.

The annual meeting of the Ohio State Board of Dental Examiners will be held at the Neil House in Columbus on the first Wednesday of December next, at 12 o'clock M., when those who desire to meet the Board will have an opportunity, and it is urgently requested that all interested be present promptly at that time.

J. Taft, Chairman.

F. H. Rehwinkel, Secretary.

### CONTENTS.

### COMMUNICATIONS.

### THE DENTAL REGISTER,

A MONTHLY MAGAZINE DEVOTED TO THE INTERESTS OF THE

**DENTAL PROFESSION.**

*Terms Reduced to $2.00 Per Year. Single Copies, 25 Cents.*

**EDITED BY PROF. J. TAFT, D.D.S.**

AND

***Published by SPENCER & CROCKER, Ohio Dental Depot.***

The volume commences in January and closes in December.

The REGISTER being issued monthly is always fresh, therefore Indispensable to the practitioner who desires to keep posted up with the new inventions and improvements which are daily developed. Neither expense nor effort will be spared to give value and variety to its contents. Articles will be illustrated with well executed engravings whenever they will add to the interest or assist to explanation.

Its extensive circulation and frequency of issue make it one of the BEST DENTAL ADVERTISING MEDIUMS in the United States.

All subscriptions must begin with January or July.

Local or special notices, five lines or less, will be inserted for $3 each insertion; over five lines, 50 cts. per line.

All advertising bills payable in advance, or at furthest, after the issue of the first number containing the advertisement. All transient advertisements to be paid for in advance.

☞CONTRIBUTIONS SOLICITED.

Exclusively Editorial Communications should be addressed to the Editor.

The latest number of the REGISTER may be ordered with or without other goods at any time, and all letters should be addressed to

**SPENCER & CROCKER, Ohio Dental Depot, Cincinnati, Ohio.**

